# The BASALT Research Program: Designing and Developing Mission Elements in Support of Human Scientific Exploration of Mars

**DOI:** 10.1089/ast.2018.1869

**Published:** 2019-03-06

**Authors:** Darlene S.S. Lim, Andrew F.J. Abercromby, Shannon E. Kobs Nawotniak, David S. Lees, Michael J. Miller, Allyson L. Brady, Matthew J. Miller, Zara Mirmalek, Alexander Sehlke, Samuel J. Payler, Adam H. Stevens, Christopher W. Haberle, Kara H. Beaton, Steven P. Chappell, Scott S. Hughes, Charles S. Cockell, Richard C. Elphic, Michael T. Downs, Jennifer L. Heldmann

**Affiliations:** ^1^ Bay Area Environmental Research Institute (BAERI), NASA Research Park, Moffett Field, California.; ^2^ NASA Ames Research Center, Moffett Field, California.; ^3^ NASA Johnson Space Center, Houston, Texas.; ^4^ Deparment of Geosciences, Idaho State University, Pocatello, Idaho.; ^5^ NASA Kennedy Space Center, Florida.; ^6^ School of Geography and Earth Sciences, McMaster University, Hamilton, Canada.; ^7^ Jacobs, NASA Johnson Space Center, Houston, Texas.; ^8^ UK Centre for Astrobiology, School of Physics and Astronomy, University of Edinburgh, Edinburgh, United Kingdom.; ^9^ Mars Space Flight Facility, School of Earth and Space Exploration, Arizona State University, Tempe, Arizona.; ^10^ KBRwyle, Houston, Texas.

**Keywords:** Mars, Spaceflight, Science, Operations, Analog, BASALT

## Abstract

The articles associated with this Special Collection focus on the NASA BASALT (Biologic Analog Science Associated with Lava Terrains) Research Program, which aims at answering the question, “How do we support and enable scientific exploration during human Mars missions?” To answer this the BASALT team conducted scientific field studies under simulated Mars mission conditions to both broaden our understanding of the habitability potential of basalt-rich terrains on Mars and examine the effects of science on current Mars mission concepts of operations. This article provides an overview of the BASALT research project, from the science, to the operational concepts that were tested and developed, to the technical capabilities that supported all elements of the team's research. Further, this article introduces the 12 articles that are included in this Special Collection.

## 1. Introduction

A human journey to Mars has long been imagined and immortalized in our collective cultural psyche by poets, writers, scientists, and technologists alike. Since the time of the Viking Missions, this momentous event has always been an elusive 30–35 years into the future; however, as this article goes to press, we find that a steep inflection in the rate of technological advancement is being met by a broad array of foundational space science and planetary research—a confluence that will optimistically serve to accelerate our path toward human exploration of Mars. Various architectures for a human journey to Mars (*e.g.*, Drake, 2009; “Journey to Mars” NP-2015-08-2018-HQ; Price *et al.*, 2015; Cichan *et al.*, 2017) include a multiple destination exploration strategy that moves human explorers from an Earth-reliant to an Earth-independent state within the next three decades. Others (*e.g.*, Musk, 2017) envision a more direct pathway to establishing a human presence on Mars. Regardless of the state of political obliquity toward landing humans on Mars, a push to get humans into deep space continues to progress and as Hubbard (2017) states “from almost any perspective, Mars is the goal for human and scientific exploration.” The question that this Special Issue addresses is, essentially, “How do we humans explore when we get there?”

In all of the mission architectures for Mars and deep space exploration, human safety will be of paramount consideration, and, as such, operational concepts and capabilities will be optimized in support of this priority as it was during humanity's last planetary excursion—the Apollo missions. However, the drive to discover and explore our Solar System will benefit from and ultimately demand the infusion of science into the operational framework and execution cadence of the mission. We contend that to enable the consideration and prioritization of science within future human planetary exploration, we must undertake this effort as a broader community and in earnest, such that from an early stage in the architecture development process we are designing the “How?” in such a way that supports both the well-being of astronauts and their ability to conduct meaningful, productive, and efficient scientific exploration.

One element of future mission design that will require rethinking with respect to the inclusion of scientific exploration as a mission priority is extravehicular activities (EVAs; defined as any space operation or activity performed outside the protective environment of a spacecraft and therefore requiring supplemental or independent life support equipment for the astronaut; McBarron and James, 1994, p.5). EVAs will be a primary mechanism for human scientific exploration within future missions; however, few EVAs dedicated to scientific exploration have ever been performed.

The quest for scientific discovery is an iterative and ceaseless process, as answers to research questions reveal more refined and sometimes unexpected research questions. In stark contrast, current EVA execution is highly scripted, and to date has been largely devoted to maintenance, installation, and construction of engineered hardware—for example, satellites and the International Space Station (Portree and Treviño, 1997)—and involves large contingents of ground-based support personnel (Miller *et al.*, 2015, 2017a); the only exceptions to this are the EVAs that occurred during the Apollo program (Neal, 2008; Miller *et al.*, 2017b).

As a whole, scientific exploration and exploratory processes have served as a secondary objective on human spaceflight missions (Love and Bleacher, 2012). As we move human exploration into deep space, EVA designs will have to balance the need for operational flight rules, technologies, and overall mission architectures that enable flexibility for scientific exploration while also ensuring operational discipline that meets heritage standards.

Another critical consideration in designing for human scientific missions to Mars is the unavoidable communication latency that will occur between Mars and Earth, ranging from 4 to 22 min one-way light time (OWLT) (8–44 min round trip). Although the planetary sciences community is experienced at conducting robotic exploration missions over these latencies (*e.g.*, Mars Exploration Rovers, Mars Science Laboratory), the tactical cadence and timelines associated with these missions (Biesiadecki *et al.*, 2006; Leger *et al.*, 2005; Grotzinger *et al.*, 2012) do not necessarily translate to the development of EVAs where, for example, intra- (within) EVA decision making between Earth and Mars teams is required. Under these communication latency conditions, ground personnel will be unable to use real-time (non-delayed) communications to support astronauts as they execute tasks or troubleshoot anomalies. Indeed, for any actions or decisions that must occur more quickly than the time it takes to complete one round-trip communication cycle between crew and the Earth, the astronauts and onboard systems will by default need to control their own situations. However, the question remains as to when and how Earth-based support could assist during EVAs.

Finally, another significant constraint in future EVA operations is the bandwidth afforded by the eventual communications architecture that will impact the ability to share data products and other communications between space and ground during EVA. Unlike communication latency, the bandwidth of communications is a mission design parameter that can be increased through additional investment in space communications technology and infrastructure; however, although few would argue against increased communications bandwidth as a desirable capability, it must compete with many other required and desired capabilities for a finite budget. Guidance and insight are still needed to inform the difficult decisions on how to invest limited resources to achieve maximum likelihood of mission success.

So how do we begin the process of redesign toward addressing these fundamental mission considerations and infusing them with science priorities? One of NASA's approaches to fulfilling this infusion of science into human mission architecture development is through “Analog Missions”—Earth-bound missions that examine scientific, operational, and technical elements that are effectively analogous to conditions on other planetary and deep space environments (*e.g.*, Lee and Osinski, 2005; Léveillé, 2010; Lim *et al.*, 2011; Reagan *et al.*, 2012; Eppler *et al.*, 2013; Rader *et al.*, 2013). These analogs vary in their purpose, from being focused primarily on science such as astrobiology and comparative planetology (*e.g.*, Perez-Chavez *et al.*, 2000; Keszthelyi *et al.*, 2004; Hynek *et al.*, 2013; Yesavage *et al.*, 2015; Payler *et al.*, 2016), operations (*e.g.*, Abercromby *et al.*, 2013a, b; Bleacher *et al.*, 2013; Chappell *et al.*, 2013; Hurtado *et al.*, 2013; Love and Reagan, 2013), or technology development (*e.g.*, Cannon *et al.*, 2007; Fong *et al.*, 2008; Glass *et al.*, 2013), to those that more overtly integrate each of these three streams (*e.g.*, Lim *et al.*, 2011; Eppler *et al.*, 2013; Heldmann *et al.*, 2016; Miller *et al.*, 2016, 2018; Kobs Nawotniak *et al.*, 2019). In the case of the latter analog type, unique and flight-relevant operational environments are hypothesized and created to examine knowledge gaps related to operations involving human scientific exploration of deep space and Mars.

The articles associated with this Special Collection focus on the NASA BASALT (Biologic Analog Science Associated with Lava Terrains) Research Program. BASALT focuses on integration—it is a science-driven mission conducted under Mars mission conditions—and the project exists to both broaden scientific knowledge regarding the habitability of basalt-rich terrains on Mars and move the pendulum from Earth-reliant to Earth-independent human exploration through the examination of the effects of science on current Mars mission concepts of operations (ConOps) and through the examination of these effects on the operational culture of current human spaceflight missions. Our science is anchored in the investigation of terrestrial volcanic terrains and their habitability as analog environments for early and present-day Mars, which necessitates the inclusion of field work at strategically selected locations on Earth. However, what distinguishes BASALT from typical terrestrial field programs is that our fieldwork is conducted under simulated Mars mission conditions. Specifically, BASALT's focus is on the iterative development and evaluation of capabilities and ConOps to enable efficient and effective cooperation between scientific explorers on Mars and support personnel on Earth, and it is focused on the more challenging problem of enabling meaningful scientific cooperation throughout execution (intra-EVA) rather than solely the periods between EVAs (*i.e.*, inter-EVA).

Human mission concepts that effectively mirror current robotic architectures or that invoke command-and-control hierarchies may result in mission concepts that limit science discourse between the Mars crew and the Earth-bound Mission Support Center (MSC) to inter-EVA periods. Although inter-EVA space-ground communication will be of significant value to both science and operations, an inability to communicate intra-EVA would mean that Earth-based inputs would be limited to strategic communications, leaving tactical decisions entirely to the crew. The crew will undoubtedly be well trained in future missions; however, it is unlikely that they will be the subject area experts (SAEs) in the multitude of scientific fields planned for future missions; the true SAEs will be located on the Earth in MSCs. In addition, these scientific disciplines will likely require a breadth of science teams, all competing for their scientific objectives to be prioritized and satisfied. The management and organization of these scientific teams will need careful thought and consideration, especially when we deal with human-scale operations. Even with communications latency, and perhaps even because of that latency, the pace of scientific EVA operations will be much greater than ever before. Thus, it is critical to evaluate the viability of intra-EVA communications ConOps that allow SAEs to influence scientific and exploration decision making on Mars.

Many questions remain as to whether these intra-EVA communications can be conducted under Mars–Earth latency conditions, and whether they are enabling and enhancing to the science return. If SAEs are to be leveraged in scientific exploration, they must be supported to achieve the highest possible scientific input. A primary challenge in enabling meaningful and efficient space-to-ground cooperation during EVAs is to figure out how to ensure that the Earth-based SAEs are provided adequate time to receive, view, assimilate, and act on data that are collected during an EVA while minimizing or eliminating the need for EV crewmembers to retrace their steps or stand idle while waiting for the resulting recommendations to reach them. Such is the challenge that the BASALT program undertook.

## 2. BASALT Work Environments

To create the BASALT research “stage” within which we could address said challenges, we had to build a truly interdisciplinary environment that enabled the BASALT team to merge scientific, operational, and technical research objectives. This necessitated a team who willingly explored a variety of solutions, many outside their traditional intellectual comfort zones, to ensure that all aspects of the research program were given their due attention to meet stated objectives. This BASALT team divided their time between two high-level work environments: (1) In-Field and (2) Out-of-Field (X-Field). In-Field and X-Field activities comprise ∼9% and 91% of our team's annual efforts, respectively. Although the In-Field component of the program typically takes center stage given the intensive travel, logistics and outreach activity associated with fieldwork, it is important to note that the bulk of the research, planning and development efforts occur outside (X-Field) of this more visible snapshot of our program.

**FIG. 1.**
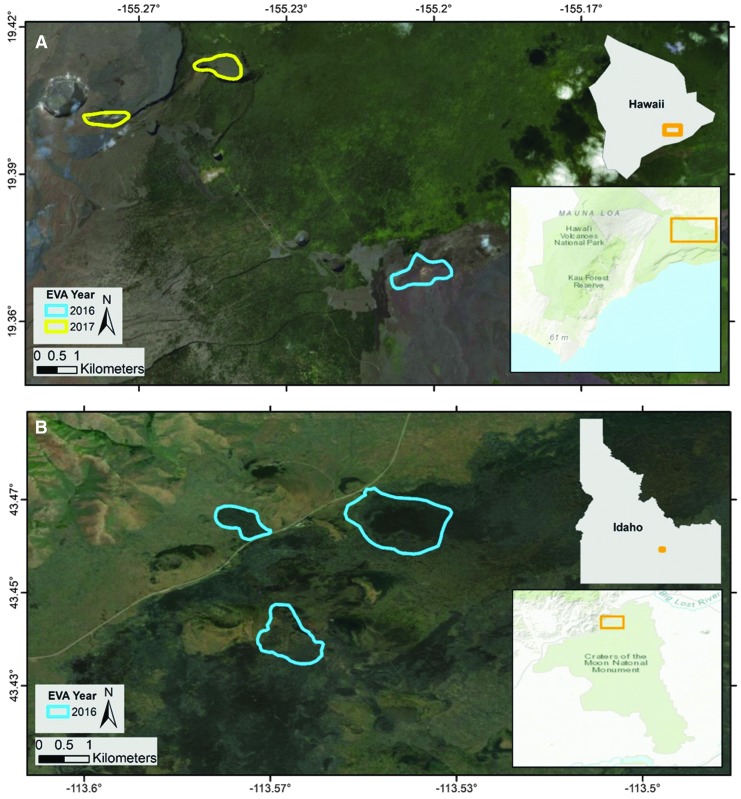
**(A)** Big Island, Hawai‘i, with locations of 2016 (blue) and 2017 (yellow) regions of interest (clockwise from top left: Keanakakoi, Kilauea Iki, and Mauna Ulu); these regions were within the Hawai‘i Volcanoes National Park. All EVA stations were found within each of these areas. **(B)** Eastern Snake River Plain, Idaho, with locations of 2016 (blue) regions of interest (clockwise from top left: Highway Flow, North Crater Flow, and Big Craters Flow); these regions were within the Craters of the Moon National Monument and Preserve.

The In-Field activities have included (1) three BASALT field deployments lasting 3 weeks (inclusive of set-up and de-mobilization periods, and a nominal 10-day mission simulation), along with (2) a number of smaller reconnaissance, engineering, and operational readiness tests that in some cases took a portion of the team into field settings. In the case of the longer (3 weeks) In-Field deployments, these took place in the Eastern Snake River Plain (ESRP) Craters of the Moon National Monument and Preserve (COTM), Idaho, from June 13 to July 1, 2016 (BASALT-1), Hawai‘i Volcanoes National Park, Mauna Ulu Region, Hawai‘i, from November 1 to 18, 2016 (BASALT-2), and Hawai‘i Volcanoes National Park, Kilauea Iki and Kilauea Caldera Regions, Hawai‘i, from November 2 to 19, 2017 (BASALT-3) (Fig. 1).

The BASALT team selected these field sites given their representation of specific paleo- and present-day states on Mars and to address the team's broader science question: How do microbial communities and habitability correlate with the physical and geochemical characteristics of chemically altered basalt environments? These terrestrial basalt environments were targeted as Mars analogs to investigate whether particular geochemical and petrological conditions could provide appropriate energy sources, major biogenic elements (CHNOPS), liquid water, and micro-habitats for microbial growth. Further, we investigated the presence of organisms using redox couples shown to exist on Mars and that have been proposed as the basis of potential chemolithotrophic ecosystems (Grotzinger *et al.*, 2014).

The complementary nature of the Idaho and Hawai‘i basalt environments provided an opportunity to conduct field research that would enable the BASALT science team to directly address questions 1A, 1B, and 2A–2C presented in Table 1. Specifically, the COTM in Idaho, and volcanic flows along the East Rift Zone (ERZ) on the Big Island of Hawai‘i were selected to represent, respectively: (1) recent Mars (when basaltic volcanism was infrequent and most evidence for volcano-driven hydrothermal activity is relict), and (2) early Mars (particularly Hesperian, when basaltic volcanism and interaction with water were widespread).

**Table 1.** Biologic Analog Science Associated with Lava Terrains Research Matrix

*Science*	*How do microbial communities and habitability correlate with the physical and geochemical characteristics of chemically altered basalt environments?*	
	Geology	1A. What are the geochemical, mineralogical, and textural properties associated with basalts affected by liquid water, intrinsic volatiles, and fumarolic gases at complementary Mars analog sites?
		1B. What geochemical and petrological conditions provide appropriate energy sources, major biogenic elements, liquid water, and microhabitats for microbial growth?
	Biology	2A. What is the relationship between the physical characteristics and geochemistry of Mars analog basalts and the biomass that they can support?
		2B. What are the upper bounds on the biomass that could have been supported on Mars?
		2C. How does the upper bound inform future requirements to detect extinct life on Mars?
*Science operations and technology*	*Which exploration ConOps and capabilities enable and enhance scientific return during human exploration activities under Mars mission constraints?*	
	Science operations, technology, and science support capabilities	3A. Do the baselined Mars mission ConOps, software systems, and communications protocols developed and tested during previous NASA analog tests work acceptably during real scientific field exploration? What improvements are desired, warranted, or required?
		3B. Do these ConOps, software systems, and communications protocols remain acceptable as communications latency increases from 5 to 15 mins OWLT? What improvements are desired, warranted, or required?
		3C. Which capabilities are enabling and significantly enhancing for Mars scientific exploration?
		3D. Do these capabilities remain enabling and significantly enhancing as communication latency increases from 5 to 15 mins OWLT?
		3E. Do these capabilities for Mars scientific exploration remain enabling and significantly enhancing as communication bandwidth decreases?

ConOps = concepts of operations; OWLT = one-way light time.

The scientific rationale for these studies stems from the hypothesis that widespread basaltic volcanism occurred on Mars through the early Hesperian, with lesser, more localized volcanism through the Amazonian period. The underlying hypothesis here is that these volcanic environments could have led to the creation of habitable environments (Werner, 2009). There is compelling evidence in orbital imagery and spectral data for the interaction between basaltic volcanism and ground ice and water over a wide range of physical scales (Squyres *et al.*, 1987; Gulick, 1998; Schulze-Makuch et al., 2007; Dohm *et al.*, 2008; El Maarry et al., 2012; Scanlon *et al.*, 2014). Further, the *Spirit* rover has provided compelling *in situ* geochemical evidence for volcanically driven hydrothermal activity, particularly at Home Plate (Schmidt *et al.*, 2008; Squyres *et al.*, 2008).

However, the question remains as to whether these hydrothermal environments on Mars were habitable. Perhaps the most fundamental factor relevant to the physical and chemical characteristics of microenvironments, and therefore their habitability, is the interaction of substrates and volatiles. The COTM and ERZ flows present complementary basalt environments affected by liquid water, intrinsic volatiles, and fumarolic gases with a range of alteration products. COTM has a geologically young basaltic terrain (eight eruptive episodes between ∼15,000 and 2000 years before present) that provides targets to observe a myriad of alteration states of basalt (including volatile interaction and subsequent weathering), and it is geologically analogous to the low-shield fields and lava plains on Mars (Greeley, 1977, 1982). Our field targets within ESRP are relatively young, chemically diverse basaltic lava flows erupted from fissures and low shields (Kuntz *et al.*, 1992; Hughes *et al.*, 1999, 2002). By comparison, the basaltic terrains on the Big Island of Hawai‘i provided a range of volcanic features that complement those found on the ESRP (Ellis, 1825; Dutton, 1884; Nichols, 1939; Wentworth and Macdonald, 1953). The historically active volcanoes, such as Kilauea, enabled the investigation of relatively sterile, recently erupted lava as well as basaltic substrates and hydrothermal steam vent environments (fumaroles) that have developed microbial habitats during historical times, and that may be important analogs for past microbial habitats on Mars (Schiffman *et al.*, 2006).

BASALT In-Field science was accomplished through geological and biological sampling and *in situ* interrogations of the basalts in Idaho and Hawai‘i conducted under simulated Mars mission conditions. Specifically, the In-Field environment comprised two working conditions: (1) In-Simulation (In-Sim) and (2) Out-of-Simulation (X-Sim). Both In-Sim and X-Sim elements were required to support the project's focus on science initiatives and the iterative development and evaluation of capabilities and ConOps to enable efficient and effective cooperation between scientific explorers on Mars and support personnel on the Earth. These ConOps and capabilities were developed from previous NASA trade studies and analog testing (NASA, 1972; Hodges and Schmitt, 2011; Lim *et al.*, 2011; Yingst *et al.*, 2011; Eppler *et al.*, 2013; Chappell *et al.*, 2016; Miller *et al.*, 2016; Beaton *et al.*, 2017), where the purpose was to understand the operational implications and interdependencies of various mission architectures being considered by NASA for future exploration missions. BASALT was an opportunity to test those ConOps and capabilities that were previously measured as enabling or enhancing within a science-driven setting where meeting peer-reviewed pressures was not simulated.

**FIG. 2.**
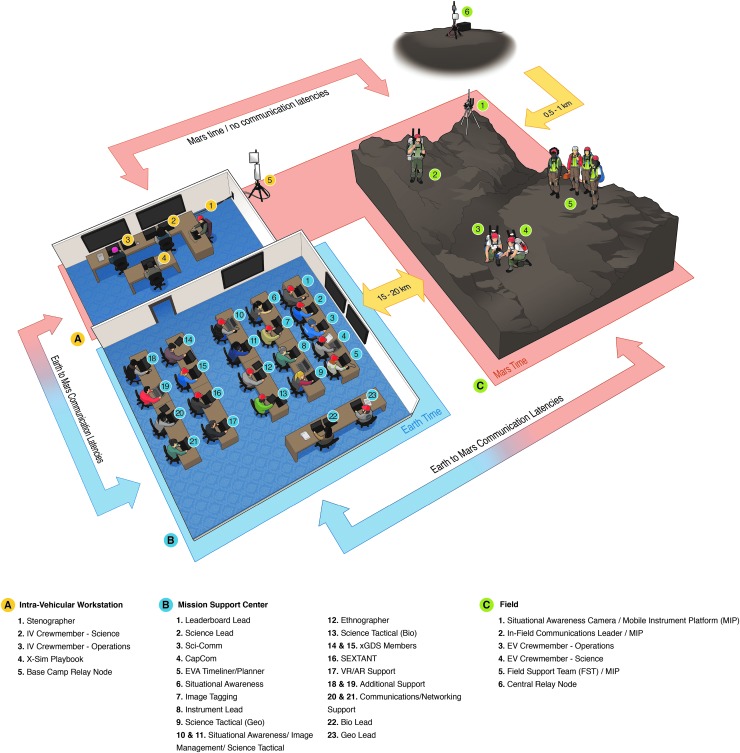
BASALT In-Field Mission Architecture for BASALT-2 and BASALT-3 deployments to Hawai‘i. The IV and MSC teams were located in the Lehua Room conference facilities at the Kilauea Military Camp; however, these teams were physically separated by a closed door during the In-Sim activities given that the IV crewmembers were on “Mars time” with the EV crewmembers, whereas the MSC was on “Earth time.” The Sci-Comm and CapCom personnel who were situated in the MSC lead all communications with the IV team members over simulated Mars latency conditions. The IV workstation included laptop computers, tablets, and additional wall-mounted display screens for each IV crewmember. Audio headsets with push-to-talk capabilities were used for voice communication. The MSC consisted of three rows of tables to accommodate individual laptops for all MSC members; two additional display screens were used at the front of the MSC room to project timeline, video imagery, and telemetry data; one additional screen on the far left wall projected dynamically updating leaderboard data to the entire SST. Network connectivity from the EVIBs and mobile SA camera to the IV workstation and MSC was enabled through the use of fixed antennae and mobile repeaters located between the field sites and the MSC. The BASALT-1 deployment to Idaho followed a similar set-up, with the major variant being that in Idaho an MMCC (trailer) was used to house the MSC and IV teams. BASALT, Biologic Analog Science Associated with Lava Terrains; EV, extravehicular; EVIB, extravehicular informatics backpack; IV, intravehicular; MMCC, Mobile Mission Command Center; MSC, Mission Support Center; SA, situational awareness; SST, Science Support Team; VR/AR, virtual reality/augmented reality; xGDS, Exploration Ground Data Systems.

To create a “flight-like” In-field test environment, the BASALT team examined analytical assessments and lessons learned from previous NASA trade studies and analog missions, and from there chose to adopt a Mars mission architecture that simulated a crew of four colocated on Mars in the roles of extravehicular (EV) and intravehicular (IV) personnel, and an MSC on Earth that comprised science (termed Science Support Team or SST) and capabilities support personnel. Table 2 provides a list and a brief description of each role within the adopted mission architecture, whereas Figure 2 outlines the baseline In-Field architecture associated with our BASALT 1–3 deployments. All of these details are expanded on throughout this Special Collection.

**Table 2.** BASALT In-Sim Key Roles and Functions

Mars-based crews	*2 EV crewmembers*: in the field cooperatively completing the science and exploration tasks associated with an EVA, while interacting with the IV crewmembers; EV-1 is the operations crewmember and leads timeline management, traverse navigation, and other operational tasks, whereas EV-2 is the science crewmember and leads all matters associated with science execution and decision making in the field.
	*2 IV crewmembers*: inside an IV workstation guiding the EVAs; IV-1 is the operations lead, and it primarily interacts with the EV crew and MSC (via CAPCOM) on operational tasks, timelines, constraints, and procedures, whereas IV-2 is the science lead, and it primarily interacts with the EV crew and MSC (via SCICOM) on science tasks, priorities, and recommendations.
Earth-based MSC	*Flight director*: has authority over all operational inputs from the MSC.
	*CAPCOM*: communicates with IV-1 on operational tasks, timeline, constraints, and procedures.
	*SCICOM*: communicates with IV-2 on science tasks, priorities, and scientific inputs from SST; tracks EVA timeline; and keeps SST apprised of critical bingo times that affect decision making based on current communication latency.
	*EVA planner*: monitors and updates timeline based on EV crew progress; assists SCICOM and CAPCOM with tracking critical bingo times.
	*SST*: includes all personnel directly supporting science associated with EVAs. SST members also actively participate in all pre-mission planning and post-mission scientific analyses.
	*SST lead*: has management authority over all scientific input and recommendations from the SST and as warranted, MSC, in general; leads SST in providing tactical feedback to EV/IV crew.
	*Biology lead*: provides coordinated feedback to SST lead regarding features that may have an impact on habitability and/or the microbial community.
	*Geology lead*: provides coordinated feedback to SST lead regarding geological features.
	*Instrument lead*: examines the scientific instrument data and offers input to SST based on the instrument scans.
	*Image tagging*: carefully examines the details of the incoming still imagery and tags them with contextual information (*e.g.*, station number).
	*Leaderboard lead*: records the science priorities, alternatives, and rationale based on SST discussions.
	*Situational awareness/image management*: keeps track of the location of the EV crew and where they are in the EVA timeline.
	*Other SST participants*: SAEs that work with the science leads to tactically and strategically plan and guide EVA execution.

EV = extravehicular; EVA = extravehicular activity; IV = intravehicular; MSC = Mission Support Center; SAEs = subject area experts; SST = Science Support Team.

**FIG. 3.**
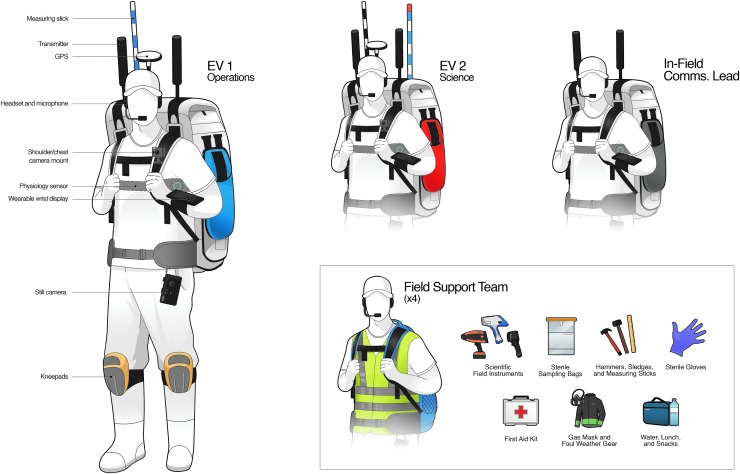
In-Sim and X-Sim EVA Personnel. EV-1 Operations wore an EVIB color-coded with blue pockets, whereas EV-2 Science wore an EVIB color-coded with red pockets. Both EV crewmembers had similar gear though their roles were focused on specific tasks that were more operationally or scientifically oriented. The Communications Lead oversaw all network and communications elements in the field, and they wore an EVIB color-coded with silver pockets. The Communications Lead's EVIB acted as an important network relay node during EVAs. Four to five FST members assisted with each EVA, though in an X-Sim capability. They acted as MIPs for the EV crewmembers and played a key role in managing handheld instruments, sterile sampling tools, safety equipment, and food/water for the entire field team, including the EV crewmembers. FST, Field Support Team; MIP, Mobile Instrument Platform.

As shown in Figures 2 and 3, both In-Sim and X-Sim elements were required to support each EVA. X-Sim elements were used where the objectives of the test did not require that those functions be performed In-Sim. For example, because surface mobility systems were not being evaluated, the transportation and positioning of various supporting field assets such as communications relay stations and sampling equipment were performed by X-Sim personnel, which included a Field Support Team (FST) and others in the role of Communications Lead and Relay Support (Fig. 2), where in reality it would most likely be transported on a motorized chassis, also known as an MIP (Mobile Instrument Platform). MIPs have been conceptualized as a vehicle that could range from a small unmanned robot to a large pressurized or unpressurized human-rated vehicle but which, in all cases, consists of a mobility system combined with a minimum set of capabilities that are relevant to science and science operations.

All EVAs were conducted under simulated Mars mission (*i.e.*, In-Sim) conditions involving four different and realistic Mars-to-Earth communication test conditions: 5- and 15-min OWLT communication latencies and low (0.512 Mb/s uplink, 1.54 Mb/s downlink) and high (5.0 Mb/s uplink, 10.0 Mb/s downlink) bandwidth conditions that represent two alternative technical communication capabilities currently proposed for future human exploration missions (Beaton *et al.*, 2017). The EVAs were optimized in their phasing, tasking, and conditioning to support the highest degree of scientific return and productivity under these In-Sim conditions. In addition, specific capabilities (including software and hardware elements) were tested and applied during these EVAs to both support science return for the BASALT team and evaluate their ability to enhance or enable our science.

During each of the BASALT field deployments (BASALT 1–3), there were 10 mission days, with daily 4 h science-driven EVAs. Each EVA involved the entire MSC and a rotating roster in the roles of EV crewmembers and IV crewmembers. The EV- and IV-crewmember rotations were structured such that each pairing would be exposed to the varying In-Sim test conditions twice or more, but to an equal number; further, the rotations enabled crewmember rest days and the ability for them to participate in the MSC.

**FIG. 4.**
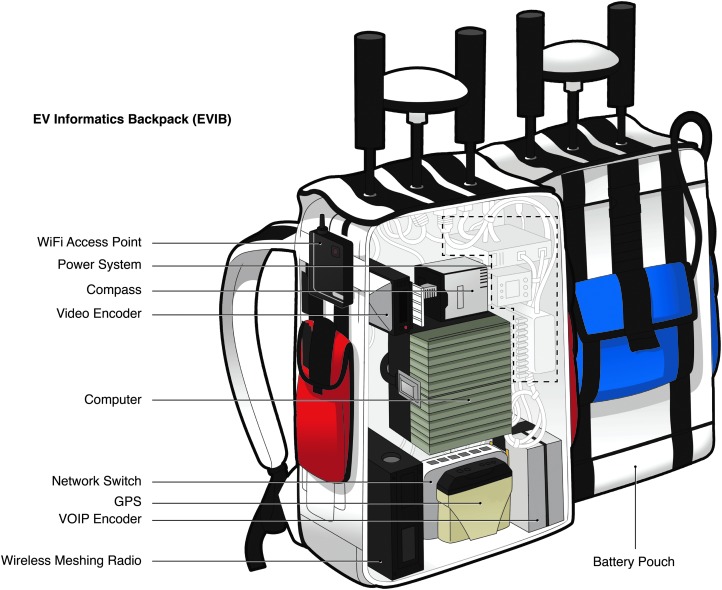
Internal hardware elements of EVIBs.

**FIG. 5.**
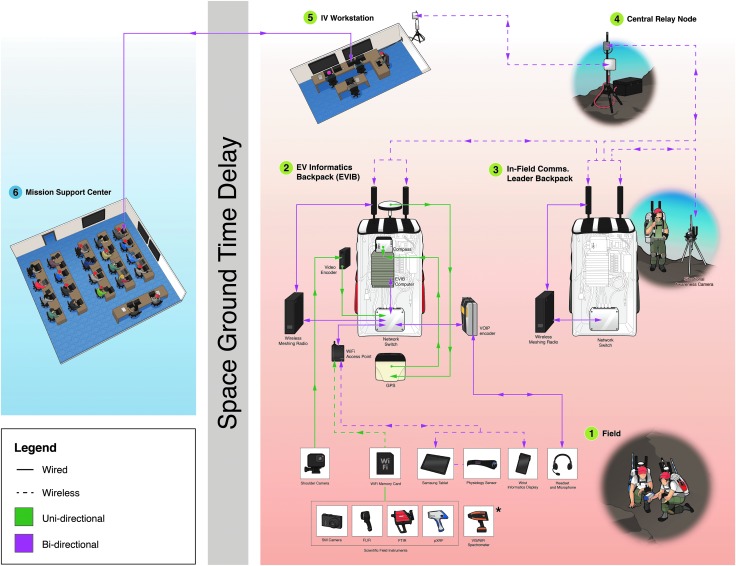
BASALT In-Field communications architecture. Fuchsia arrows represent full-duplex (two-way) data transmission, while green arrows represent unidirectional/one-way data transmission flows. Voice communications were transmitted and received by using a Voice over Internet Protocol (VoIP) system that converted analog audio from the EV crewmember headsets to digital audio for transmission over the network. Digital video from the chest cameras was transcoded in real time by a Teradek Cube encoder into a format that was suitable for streaming over the field network. Data from the still cameras and science instruments were sent via a WiFi SD card to the EVIB computer, where they were buffered in case of network dropouts and then forwarded over the network to servers in the IV Room and MSC. Similarly, GPS and compass data were collected by the EVIB computer and converted to a format that was suitable for sending over the field network by using the open-source gps2udp software package. *During In-Sim activities, the VIS/NIR spectrometer data was sent from the field to the MSC by capturing a still photo of the display screen output and sending the image via WiFi SD onwards per earlier description.

During each EVA, there were two In-Sim EV crewmembers, one situational awareness camera, and five to six X-Sim personnel in communications and FST (Figs. 2 and 3). Each EV crewmember and one additional X-Sim communications support person wore an extravehicular informatics backpack (EVIB), which was designed in support of our science and operations research requirements to represent an element of the (In-Sim) MIP capability. The EVIBs performed as critical data capture, conversion, and relay nodes within the BASALT In-Field network architecture (Figs. 3–5). Similar EVA backpacks were utilized during the NASA Desert Research and Technology Studies field tests (Abercromby *et al.*, 2013a) and when training crewmembers during the Apollo program to provide the functional capabilities of an exploration EVA suit's communications, data collection, and information systems without the cost, complexity, and excessive mass of using a pressurizable EVA suit prototype or EVA-compatible interfaces. The objectives of the BASALT project did not require a pressurized planetary spacesuit prototype; indeed, the physiological demands of working inside a heavy pressurized spacesuit in Earth gravity would have significantly and unrealistically impaired the ability of the simulated EVA crewmembers to perform basic EVA tasks such as walking. The physiological and ergonomic constraints and considerations associated with working in pressurized spacesuits is studied separately by members of the BASALT project team in simulated reduced gravity environments at the NASA Johnson Space Center (Abercromby, 2017). Tasks performed during BASALT field testing were consistent with the capabilities of test subjects working in pressurized planetary prototype spacesuits when working under reduced gravity conditions.

As shown in Figure 3, the EVIBs were color-coded to represent the EV operations lead (Blue), EV science lead (Red), and X-Sim Comms lead (Grey). The soft shell of the EVIBs was constructed from multiple types of high-tech Dyneema fabric, and it was custom designed and manufactured by Mission Workshop San Francisco in collaboration with the BASALT team. The custom elements included ports for hardware connectors associated with our communications network, a zippered compartment for rapid battery swaps, mounts for the EV video camera, and rain covers to protect sensitive equipment during frequent rain events in the field. The FST wore standard 50–80 L backpacks that carried biological and geological sampling equipment, various portable handheld scientific instruments, safety equipment, foul weather gear, and water and food for the field team (Fig. 3). As the EVA progressed, the FST handed off and retrieved equipment from the EV crew, thereby simulating one of the intended functions of a MIP, which in our application was a scientific EVA tool and sample stowage capability. Among many essential tasks, the FST was also responsible for managing and storing all field samples, and for transporting them out of the field and back to the BASALT basecamps. The EV crew members were heavily loaded with both equipment (*e.g.*, 20–22 kg/45–50 lbs EVIBs) and science/operations tasks; as such, FST assistance was critical in ensuring that the EVAs could progress reasonably from both a safety and logistics standpoint.

Another key role of the EVIBs was to house various hardware components that enabled data products (*e.g.*, telemetry, voice, video, portable instrument data) to stream from the field to the MSC and for certain products to flow in a full-duplex manner between the EV/IV and MSC (Figs. 4 and 5). All of the EVIB devices were wired to a network switch in the backpacks where data could be encoded and then carried over an internet protocol (IP) network implemented with a wireless meshing network local to the field site and two long-range fixed network links to allow transmission between the EV/IV crewmembers and the MSC (Figs. 2, 4 and 5). For example, in Hawai‘i, the first link was between a mountaintop overlooking the field site and a receiving antenna located at the United States Geological Survey (USGS) Hawai‘i Volcano Observatory (HVO). The second fixed link transferred data from HVO to the IV and MSC facility where standard wired and wireless networking was used to connect the IV and MSC computers to the field network. As shown in Figure 4 and described in the associated caption, hardware components in the EVIBs converted data and communications to and from the field into a format that was suitable for sending over the IP network.

The BASALT SST, which comprised junior and senior research contributors, was a core component of the MSC. The MSC also included X-Sim personnel in various technical, logistics, and management roles (Table 2; Figs. 2 and 3). During BASALT-1, the MSC was located within the NASA Mobile Mission Command Center (MMCC) trailer (Lim *et al.*, 2011, Figure 18) that was positioned in Arco, Idaho, nearly 50 km away from the EVA field site; during the BASALT-2 and BASALT-3 deployments, the MSC was located at the Kilauea Military Camp (KMC) that was near the north rim of Kilauea Caldera and ∼5–15 km away from the Hawai‘i field regions. These MSC locations were selected based on technical and logistical rationale, and they also served as “Base Camp” for the entire team—that is, the area where all personnel mustered at the end of the day for food, housing, and meetings. Figure 2 also shows that though in reality the IV crewmembers would be colocated on Mars (either in orbit around Mars or on the surface of Mars) with the EV crewmembers, during the BASALT EVAs the IV crewmembers were stationed in an isolated room in either the MMCC (Idaho) or KMC (Hawai‘i) to facilitate access for X-Sim hardware and software support personnel. However, a broad wireless network connecting the MMCC/KMC to the field enabled the IV crew to have full-duplex (bi-directional) no-latency interactions with the EV crew on “Mars time,” and full-duplex communication under Mars–Earth latency conditions with the MSC (Fig. 2).

## 3. BASALT Special Collection Compendium

The BASALT research program relied on the scientific, operational, and technical elements previously described to function as an integrated whole during each of the In-Field deployments. The articles within this Special Collection represent a portion of the BASALT program's research output, and they delve into the results and details associated primarily with the BASALT-1 and BASALT-2 deployments. Some of the articles include analysis and discussion from BASALT-3, with additional results from that deployment presented in forthcoming publications.

Hughes *et al.* (2019) begin our compilation with an overview of the field areas in Idaho and Hawai‘i selected for study in BASALT. Particular focus is given to the various alteration states of volcanic rocks within the field areas, their value as scientific analogs for martian sites, and their use for constraining the habitability potential of various basaltic substrates. Geologic descriptions of the research field areas, presented as rationale for selection as Mars analogs, emphasize compositional diversity in rock types and the differences in climate related to their geologic settings in oceanic and continental regimes. Mars' geologic history and the implications of early- and present-day climate on Mars are discussed to demonstrate that our selection of field research areas helps to further understand the connection between geologic substrates and biological activity.

Next, Cockell *et al.* (2019) present an examination of the biomass and diversity of life in basaltic terrains on the Earth that are exposed to transient meteoric and magmatic aqueous alteration as a means to understand whether related Mars environments could have hosted life and what biomass was sustainable. Specifically, Cockell *et al.* quantify the biomass and diversity of life in basaltic features that focused on active and inactive fumaroles and unaltered and meteorically altered basalt from the Hawai‘i and Idaho field sites. The results Cockell *et al.* obtained lead to general conclusions about the biological potential of martian basaltic terrains, and observations about what these results might imply for the human exploration of Mars.

The Collection then transitions to a focus on Science Operations. Beaton *et al*. (2019a) provide a background on the foundational elements of the BASALT Operations and EVA research, including details on the crewmember selection rationale, and an overview of the rigorous assessment methodology used to evaluate the enabling and enhancing aspects of various ConOps and Capabilities that the BASALT team tested in support of an acceptable level of science return during human Mars missions. This article examines community best practices that derive from heritage spaceflight culture and those that have been identified through recent analog mission tests, while also presenting new best practices that have been identified through the science-driven Mars mission simulations of the BASALT project. Beaton *et al.* report that even with Mars communication latencies and bandwidth constraints, it is possible to have meaningful tactical and strategic interaction with an expert science team on Earth during both intra- and inter-EVA periods, and that the impact of these working conditions on EVA efficiency and science return can be largely mitigated through the planning and communication techniques and capabilities developed and tested through the BASALT research program.

Building on Beaton *et al. *(2019a), Beaton *et al.* (2019b) detail the subjective measures of acceptability and capability assessment used to establish the level of acceptability for the baseline ConOps and the level of mission enhancement provided by the ConOps' baseline capabilities. This article provides ConOps and capability recommendations for future analog research and future human Mars exploration missions.

Brady *et al.* (2019) examine the translation of traditional, single-discipline field research strategy on Earth to multidisciplinary, large-team approaches needed for planetary exploration. Beginning with a broad Science Traceability Matrix (STM), Brady *et al.* (2019) illustrate how a multidisciplinary science team developed strategies for distilling scientific hypotheses into specific EVA objectives, reached consensus on observational and instrumental data products critical to enabling successful sample collection, and identified limitations of available precursor data used in EVA planning that may be used to inform future planetary missions.

Stevens *et al.* (2019) discuss the intra-EVA tactical scientific decision-making that the BASALT team carried out on both sides of the Earth–Mars divide in order to achieve the strategically defined scientific objectives. They offer suggestions for procedures that allowed clear communication and successful scientific returns within the BASALT ConOps, including assessment of still photography as one of the most powerful capabilities used by the SST to evaluate proposed sample locations. Critically, they describe the ways in which the SST was able to provide useful tactical guidance to the IV/EV crewmembers throughout an EVA, demonstrating that this ConOps may be a powerful tool to enhance scientific exploration on Mars despite communication latency.

Payler *et al.* (2019) explore the evolving roles and organization of the SST in the MSC as it adapted to meet the challenges of In-Sim support of the Mars-based EV/IV crew. Some adaptations focused on the physical layout of the team to improve within-team discussion and consensus, whereas other challenges required the SST to create new personnel positions to better manage incoming and outgoing information. A key finding is the repeated tendency of the SST to reject hierarchical, pyramid-shaped physical layouts in favor of collaborative roundtables, even as they committed to increased task differentiation and structured roles. Inherent in their findings is the necessity of staffing the SST, and the entire MSC, with those who are prepared to be collegial, resourceful, and goal oriented throughout the mission. Constant and timely scientific feedback was prioritized during the BASALT EVAs, and, as such, the operational cadence of the mission was steeped with urgency. This intensity had to be balanced with the need to provide thoughtful, rigorous, and systematic scientific input to the EV and IV crewmembers to optimize each decision for the highest degree of scientific productivity.

Sehlke *et al.* (2019) move the Collection into a focus on scientific capabilities in support of future human planetary missions. Specifically, they explore the use of portable handheld spectrometers during EVAs. The incorporation of these types of scientific capabilities has been envisioned for future human planetary missions; however, questions remain in the literature regarding their ability to enhance or enable scientific decision-making during both intra- and inter-EVA periods. As well, Sehlke *et al.* (2019) examine specific guidelines and technical requirements associated with effectively incorporating these instruments into human missions to Mars and pursuing a path to develop a “geological tricorder” for future astronauts.

Kobs Nawotniak *et al.* (2019) examine the modes of communication between the Mars- and Earth-based teams, discussing the relative uses of video, still imagery, audio, and text-based communication employed by the BASALT team. Most of these modes carried information from EV/IV crew to the SST, whereas text messaging was uniquely used to carry recommendations, questions, and guidance from SST back to the Mars-based crew. Communication latencies meant that there was limited, or no ability to directly fix failures in communication, and any failure was likely to have an impact on the efficiency of the EV crew. Kobs Nawotniak *et al.* (2019) consider the shorthand communication developed by the team over the course of the BASALT deployments, as well as the time-variable rate and length of messages, and how the text-based communication protocols adopted by the SST enabled the IV crew to independently identify and tactically respond to unexpected failures in communication.

Marquez *et al.* (2019) discuss BASALT integration of a suite of complementary science operations capabilities that are referred to collectively as *Minerva* (after the Roman Goddess of wisdom). *Minerva* is a combination of xGDS (Exploration Ground Data Systems)—a set of tools to support science and mission operations and post-operation data analysis; SEXTANT—a resource-based path planning tool that optimizes traverses based on distance, time, or energy consumption; and Playbook—a scheduling and timelining software support tool. *Minerva* provided critical support during all phases of the mission, from planning and execution to managing visual, audio, and text communication streams that connected Earth- and Mars-based teams during EVAs. Based on field testing results, Marquez *et al.* (2019) discuss and emphasize the importance of rapid information integration within the system, explicit use of temporal tracking within the EVA timeline, and multimedia communication capability between team members.

Finally, the Special Collection is rounded out by two complementary articles (Seibert *et al.*, 2019; Miller *et al.*, 2019) that, respectively, examine the effects of analog research on the development of space communication architectures and the lessons learned from BASALT field deployments as they apply to the evolution of these deep space communication networks. Seibert *et al.* (2019) provide the first comprehensive overview of this topic, while Miller *et al.* (2019) provide analysis of the BASALT In-Field communications data products and volumes generated, transferred, and utilized by the EV/IV crewmembers and the MSC over the course of the field mission. Miller *et al.* (2019) then examine the implications of these results for future deep space networks.

## 4. Final Remarks

The articles in this Special Collection provide an academic look at the process of developing mission architectures for future human spaceflight, and they highlight our team's ongoing effort to create mission designs that optimize for the humans and their inherent capacity for exploration within the endeavor of human spaceflight. Achievement of this goal will require a continued and persistent push to find the operational “devil in the details”; to flush out the right questions that will lead to designs suited for the unprecedented mission demands that will come with having humans explore Mars and other deep space environments; and perhaps, most importantly, to provide a framework within which scientific exploration will have the ability to ebb, flow, and be dynamically driven both by the environment that we are exploring and by those who are exploring it first hand and millions of kilometers away.

The Apollo missions were not tasked with science as a priority, and in fact there was worry and debate within the scientific community of the time that there would be deleterious effects on space science research as a whole as a result of the budgetary and programmatic demands of the Manned Space Flight program (Compton, 1989). However, as the Apollo missions came to an end in December 1972, there was a synergy that had developed between the engineering, operations, and lunar science communities that was undeniably productive as a collective and within each of these disciplines. Recommendations were put forward by the scientific community in 1965 (NASA SP-88) detailing scientific priorities, crew selection, and training, supporting capabilities such as lunar aerial vehicles and long-range pressurized vehicles. Many of these recommendations were ultimately adopted in support of science during the Apollo missions; many of these recommendations remain topical today as we design for Mars. Fundamentally, the dialogue was productive for the Apollo missions, for its science output, and to space exploration as it continues today. As Compton (1989) remarks in his historical overview of the Apollo Lunar Exploration Missions, “No one lamented more strongly than the scientists—the cancellation in 1970 of three planned lunar exploration missions.” Healthy debates continue as we work to find a path forward to human exploration on Mars; however, it is without a doubt that the effort will best be served by the integration of science requirements early on, in the engineering and operational development process.

The selection of *Astrobiology* within which to house this BASALT Special Collection was done strategically, with the intent to reach and inspire a broader scientific audience to join and to lead other integrative studies that will move us toward human mission architectures that are imbued with operational concepts and capabilities that value and promote science and exploration at their core. The BASALT team will continue to assiduously push forward by examining different operational concepts, adding new capabilities to our support infrastructure, and addressing evolving knowledge gaps related to our understanding about the habitability of Mars. It is our hope that this first collection of findings, along with subsequent research output, will lead to new partnerships and innovations that will accelerate humanity toward Mars.

## Abbreviations Used

BASALT: Biologic Analog Science Associated with Lava Terrains
ConOps: concepts of operations
COTM: Craters of the Moon National Monument and Preserve
ERZ: East Rift Zone
ESRP: Eastern Snake River Plain
EV: extravehicular
EVAs: extravehicular activities
EVIB: extravehicular informatics backpack
FST: Field Support Team
HVO: Hawai‘i Volcano Observatory
IP: internet protocol
IV: intravehicular
KMC: Kilauea Military Camp
MIP: Mobile Instrument Platform
MMCC: Mobile Mission Command Center
MSC: Mission Support Center
OWLT: one-way light time
SA: situational awareness
SAEs: subject area experts
SST: Science Support Team
STM: Science Traceability Matrix
VoIP: Voice over Internet Protocol
xGDS: Exploration Ground Data Systems
